# Lateral hypothalamus, nucleus accumbens, and ventral pallidum roles in eating and hunger: interactions between homeostatic and reward circuitry

**DOI:** 10.3389/fnsys.2015.00090

**Published:** 2015-06-15

**Authors:** Daniel C. Castro, Shannon L. Cole, Kent C. Berridge

**Affiliations:** Department of Psychology, University of MichiganAnn Arbor, MI, USA

**Keywords:** lateral hypothalamus, nucleus accumbens, ventral pallidum, reward, motivation

## Abstract

The study of the neural bases of eating behavior, hunger, and reward has consistently implicated the lateral hypothalamus (LH) and its interactions with mesocorticolimbic circuitry, such as mesolimbic dopamine projections to nucleus accumbens (NAc) and ventral pallidum (VP), in controlling motivation to eat. The NAc and VP play special roles in mediating the hedonic impact (“liking”) and motivational incentive salience (“wanting”) of food rewards, and their interactions with LH help permit regulatory hunger/satiety modulation of food motivation and reward. Here, we review some progress that has been made regarding this circuitry and its functions: the identification of localized anatomical hedonic hotspots within NAc and VP for enhancing hedonic impact; interactions of NAc/VP hedonic hotspots with specific LH signals such as orexin; an anterior-posterior gradient of sites in NAc shell for producing intense appetitive eating vs. intense fearful reactions; and anatomically distributed appetitive functions of dopamine and mu opioid signals in NAc shell and related structures. Such findings help improve our understanding of NAc, VP, and LH interactions in mediating affective and motivation functions, including “liking” and “wanting” for food rewards.

## Overview

Research over the last several decades has implicated hypothalamic and mesocorticolimbic neural circuitry in food-related behaviors. In particular, the lateral hypothalamus (LH), ventral tegmental area (VTA) and nucleus accumbens (NAc) have long been known to be important in motivation for food and other rewards. More recently, the ventral pallidum (VP) has also been found to play important roles in eating and reward. Studies in our lab on the affective component of food reward have helped to produce insights into how these structures participate in generating the incentive motivation (i.e., “wanting”) or hedonic impact (i.e., “liking”) of palatable food rewards. Here, we describe and examine some findings that further define the roles of these areas in food reward.

## Early Years: Lateral Hypothalamus as the Feeding Center

During the 1950’s and 60’s, studies using electrolytic lesion and electrical stimulation techniques helped address two fundamental questions: (1) what brain areas are necessary for normal food intake, such that damage to that area would cause a reduction in eating behavior and food reward; and (2) what brain areas, when stimulated, are sufficient to cause increases in food intake? In 1951, Anand and Brobeck first showed that lesions of the LH area would cause intense aphagia and adipsia; animals would completely fail to eat or drink, and would starve to death if not fed intragastrically (Anand and Brobeck, 1951). These findings were extended in the following decade by Teitelbaum and Epstein, and by many other researchers (Teitelbaum and Epstein, 1962; Boyle and Keesey, 1975; Oltmans and Harvey, 1976; Schallert et al., 1977). In contrast to the aphagia caused by LH lesions, studies using electrical stimulation showed that activation of LH caused increases in food or water intake (Delgado and Anand, 1953; Mogenson and Stevenson, 1967; Coons and Cruce, 1968). These findings contributed to LH becoming known as a “feeding center” (Anand and Brobeck, 1951; Stellar, 1954).

In 1962, Teitelbaum and Epstein (1962) provided the first evidence that LH was also involved in causing the *hedonic* impact of palatable food. They found that lesions of LH caused the rats to react with intense aversive reactions, such as disgust-type gapes, to palatable, sweetened milk. These results also occurred in tandem with consistently observed aphagia. In their own words:

[The rat] actively resists having milk placed in its mouth by a medicine dropper, and it does not swallow the milk once it is there, but rather allows it to dribble out the side of the mouth. Ordinarily a normal rat does not show such behavior… This suggests that mouth contact with food and water is highly aversive to a rat with lateral lesions during this stage (Teitelbaum and Epstein, 1962, pp. 75–76).

Their finding of aversive gapes after LH lesions, in addition to aphagia, extended the role of LH to now include palatability, or the affective processing of food reward.

Complementing this hypothesis, some investigators proposed that LH electrode stimulation might enhance a hedonic taste signal to effectively make food taste better (Hoebel, 1988). This could be caused by direct LH projections to brainstem gustatory nuclei. Hedonic enhancement was suggested as a psychological mechanism for producing increases in food intake, and might also contribute to electrode self-stimulation effects. For example, Hoebel suggested that “… lateral hypothalamic stimulation is like palatable food in inducing both appetite and reward” (Hoebel, 1988, pp. 583–584). Regarding reward effects of LH stimulation, he further speculated:

Where is reinforcement? … One component of reinforcement could be in LH cells that enhance sweet taste input in the NST (nucleus of the solitary tract) … What is reinforcement? To this question we can now answer that one aspect of reinforcement could be taste-induced stimulation of LH cells which enhance the taste (Hoebel, 1988, p. 594).

In other words, LH stimulation was posited to enhance sweet taste signals as part of the mechanism of LH stimulation-induced reward.

## Taste Reactivity as a Measure of Hedonic Impact

To ascertain whether or not LH stimulation did in fact enhance a taste signal, a more direct measure of taste hedonic impact was needed, such as the affective taste reactivity test. This test measures the affective orofacial reactions that are elicited by different taste stimuli in animals and human infants. These orofacial expressions have been called “liking” reactions because they reflect the palatability of the taste (Berridge, 2000). Furthermore, many of the behaviors measured in this test are homologous across a number of mammalian species, including chimpanzees, orangutans, horses, mice, rats, and humans (Kiefer et al., 1998; Berridge, 2000; Steiner et al., 2001; Jankunis and Whishaw, 2013).

The taste reactivity test and its component affective facial expressions were first described for human infants in the early 1970’s by Steiner (1973). The test was soon adapted for rats using an intra-oral cannula administration of taste solutions by Harvey Grill and Ralph Norgren (Grill and Norgren, 1978b). Affective orofacial reactions include three classes of responses: (1) positive hedonic or “liking” reactions (e.g., tongue protrusions, paw licking) typically elicited by sucrose; (2) negative aversive or “disgust” reactions (e.g., gapes, chin rubs, face washing) typically elicited by bitter quinine; and (3) neutral reactions (e.g., passive drip, mouth movements) that are elicited by water and many other tastes that are relatively neutral, as well as by palatable (mouth movements) or unpalatable tastes (dripping) (Steiner et al., 2001).

Importantly, these orofacial reactions are not merely sensory reflexes to a sweet vs. bitter sensation, but reflect the *hedonic impact* of that taste sensation. One line of evidence for this comes from the observation that quite different taste sensations can elicit the same pattern of hedonic reactions, suggesting they have similar palatability (Steiner et al., 2001). For example, sucrose and dilute sodium chloride elicit similar positive patterns of “liking” reactions, despite being dissimilar sensations, and both are preferred over water by rats in intake tests (Grill and Norgren, 1978b; Flynn and Grill, 1988). Conversely, bitter tasting quinine or highly concentrated sodium chloride both elicit similar aversive “disgust” reaction patterns, and both are avoided compared to water (Berridge et al., 1984). Another line of evidence supporting a hedonic interpretation comes from observations that a single taste sensation can evoke quite different orofacial reactions under different conditions relevant to its palatability. For example, orofacial “liking” reactions to sucrose are increased by physiological hunger and suppressed by satiety states (termed alliesthesia), and orofacial reactions to concentrated NaCl are changed from negative “disgust” to positive “liking” by hormonal induction of salt appetite (Berridge and Schulkin, 1989; Clark and Bernstein, 2006). Learned shifts in palatability will similarly change reaction patterns to a particular taste that has been used as a conditioned stimulus, based on Pavlovian associations formed between tastes, or associations between a taste and its ensuing physiological consequence (e.g., visceral illness; caloric satiety). A dramatic example is Pavlovian conditioned taste aversion, in which sucrose or a similar sweet taste is associatively paired with nausea induced by a LiCl injection (Garcia et al., 1968). The learned aversion changes orofacial reactivity to the paired sweet taste from positive “liking” to negative “disgust” (Grill and Norgren, 1978a; Berridge et al., 1981; Spector et al., 1988). In short, affective orofacial reactions reflect the hedonic impact of a taste stimulus, and so are determined not only by the taste itself, but also by relevant physiological states and Pavlovian associations that influence its palatability. Finally, these affective reactions can also be dramatically changed by specific brain manipulations (e.g., lesions or stimulations), which help to reveal brain mechanisms of hedonic impact, as discussed below.

Importantly, we note that affective taste reactivity patterns still faithfully track hedonic impact, even in situations when other behavioral measures of palatability, such as voluntary food intake, or consummatory behavioral measures such as lick-pattern microstructure (i.e., lickometers), diverge from hedonic impact (Berridge, 2000). This divergence may occur because all appetitive tests and most other consummatory tests of palatability require the animal to engage in spontaneous appetitive approach toward the food stimulus and voluntarily ingest it. Relying upon voluntary intake or appetitive behavior and choice makes the test depend additionally on brain mechanisms of incentive motivation, in addition to hedonic impact. This is problematic, as incentive motivation and hedonic impact can sometimes change independently. By comparison, in the taste reactivity measure, a taste is delivered directly into the mouth to elicit orofacial reactions without any need for appetitive behavior or even voluntary ingestion. Several examples of divergence of lickometer/intake/choice measures vs. taste reactivity come from manipulations of mesolimbic dopamine systems, which change incentive motivation and consequently free intake and lickometer measures, but may not truly change hedonic impact as reflected in affective orofacial reactions to tastes. Such dopamine manipulations include neurotoxin 6-OHDA lesions of mesolimbic dopamine systems and pharmacological blockade of dopamine receptors. These manipulations suppress food intake, or even cause complete aphagia in the case of 6-OHDA lesions (similar to electrolytic LH lesions), and reduce lickometer measures of sucrose motivation/palatability (e.g., shorter lick bursts) (Rolls et al., 1974; Zis and Fibiger, 1975; Oltmans and Harvey, 1976; Berridge et al., 1989; Schneider et al., 1990; Smith, 1995; Higgs and Cooper, 2000). However, the same dopamine-suppressing manipulations do not alter hedonic impact, as reflected in taste “liking” patterns of taste reactivity (Berridge et al., 1989; Peciña et al., 1997, 2003; Wyvell and Berridge, 2000). That dissociation originally gave rise to the hypothesis that dopamine mediated incentive salience or “wanting” for food reward, rather than hedonic impact or “liking” (Berridge et al., 1989).

Other brain manipulations can further dissociate hedonic impact from other consummatory behaviors that do not depend on voluntary ingestion of an external food, such as intra-oral intake, or the passive swallowing of a taste substance already in the mouth. For example, lesions of central nucleus of the amygdala suppress the expression of salt appetite in consummatory intra-oral intake as well as in appetitive approach and voluntary intake tests, but do not suppress the increase in “liking” for intense NaCl taste that is reflected in affective taste reactivity patterns (Galaverna et al., 1993; Seeley et al., 1993). Under normal conditions, rats respond negatively with “disgust” reactions (e.g., gapes) to a hypertonic 3% NaCl solution and avoid consumption. However, in a salt depleted state, rats approach, consume, and even show “liking” reactions to the same salty taste (Krieckhaus and Wolf, 1968; Berridge et al., 1984; Schulkin et al., 1985). After central amygdala lesions, rats no longer seek or ingest the salty solution (Schulkin et al., 1985), and no longer increase their passive intra-oral intake when the salty solution is infused into the mouth (Galaverna et al., 1993; Seeley et al., 1993). Yet, when rats with central amygdala lesions are tested in taste reactivity, they still show an affective change in orofacial reactions from “disgust” to “liking” patterns (Galaverna et al., 1993). This dissociation indicates that the hedonic alliesthesia shift remains intact, but no longer controls any appetitive or consummatory aspect of ingestion (Galaverna et al., 1993; Seeley et al., 1993). Altogether, these studies indicate that taste reactivity specifically reflects hedonic impact, and under some conditions, captures hedonic shifts even more reliably than appetitive or other consummatory measures.

Regarding the neural substrates of taste reactivity behaviors, the basic orofacial reactions can be generated by brainstem circuitry alone (Grill and Norgren, 1978c). However, it is important to note that many hedonic modulations by psychological or physiological factors require forebrain control, suggesting that these orofacial reactions are more than simple reflexes (Grill and Norgren, 1978a; Kaplan et al., 2000; Grill and Kaplan, 2001; Grill, 2006). Decerebrate rats that have received transections at the level of the midbrain are still able to generate orofacial movements to sweet or bitter tastes, but no longer show sensitivity to hunger alliesthesia and are unable to learn a conditioned taste aversion (Grill and Norgren, 1978a; Kaplan et al., 2000). Finally, and most important, discrete forebrain manipulations sites in NAc, VP, and neocortex (e.g., drug microinjections, excitotoxic lesions or other manipulations) can powerfully enhance positive “liking” reactions to sweetness, whereas other manipulations at some of the same forebrain sites abolish “liking” reactions (described further below) (Smith et al., 2010). Thus, although the basic circuitry needed to generate oromotor facial patterns is contained within the brainstem, the forebrain imposes hierarchical control over affective aspects of these reaction patterns.

## Lateral Hypothalamus and Hedonic Impact?

To answer the question of whether or not LH stimulation did in fact enhance the hedonic impact of tastes as a neuropsychological mechanism to increase eating posed above, Berridge and Valenstein (1991) electrically stimulated LH and measured its effect on affective orofacial responses elicited by sucrose and quinine using the taste reactivity test, as well as measuring stimulation-evoked eating behavior. Using this measure of taste hedonic impact, Berridge and Valenstein (1991) found that electrical stimulation of LH completely failed to enhance positive hedonic reactions to sucrose, despite making the rats eat over four times as much food. If anything, “disgust” reactions to sweetness were increased during LH stimulation, providing evidence against the hedonic hypothesis for LH stimulated eating.

However, if LH is not able to generate a hedonic signal, why did LH lesions cause rats to respond aversively to the taste of milk, as described by Teitelbaum and Epstein? The answer actually appears to be that LH lesions *per se* do not produce aversion. Rather, damage to a nearby structure, the VP, is primarily responsible for the positive to negative shift in affective reactions. Careful mapping of lesion-induced effects on eating behavior is crucial for understanding the underlying neural circuitry (Khan, 2013). Several subsequent mapping studies following Teitelbaum and Epstein’s findings indicated that LH damage was not the cause of the “disgust” reactions. First, it is important to note the electrolytic lesions produced by Teitelbaum and Epstein (1962) were large by contemporary standards, damaging structures outside the LH, as well as the LH itself. Those lesions extended as far rostral as to include VP, and as far caudal as to include premammillary nucleus, thereby damaging VP, lateral preoptic area (LPO), subthalamic nucleus, and portions of the sublenticular extended amygdala. Morgane (1961) independently showed that large electrolytic lesions of ventral globus pallidus produced aphagia comparable to LH lesions, and while his study did not report whether aversive reactions to taste were induced by the globus pallidus lesions, it can be noted that the globus pallidus in brain atlases at the time extended ventrally into what is now recognized as VP. Further localization of function came from an anatomically-detailed study by Schallert and Whishaw (1978) which separated LH into anterior and posterior regions, and demonstrated that “disgust” induction was not simply due to damage of LH as a whole. Schallert and Whishaw showed that only in the anterior half of LH were electrolytic lesions able to cause the “disgust” reactions to sucrose taste, as well as aphagia. By contrast, posterior LH lesions only induced passive aphagia without inducing any active aversion or disgust to palatable foods. In our view, inspection of the histology figures of Schallert and Whishaw suggests that their anterior LH lesions also extended rostrally into the VP and the LPO (pg. 736, Figure 7).

To more thoroughly localize the site of “disgust” release, Cromwell and Berridge (1993) made smaller excitotoxic lesions, comparing lesion sites in VP, rostral LH, LPO, and nearby regions of striatum, globus pallidus, extended amygdala, or substantia innominata. Cromwell mapped consequent changes in motivated food intake and hedonic taste reactivity on to the sites of damage, and found only lesions that damaged VP produced the change in affective responses from “liking” to “disgust”. That is, even anterior LH/LPO lesions did not produce greater numbers of “disgust” reactions to the taste of sucrose if VP was spared, even though aphagia was produced by all lesion sites in LH, LPO, and VP. Later studies found that transient inhibition of VP neurons via hyperpolarizing muscimol microinjections was enough to cause aversive “disgust” reactions to sucrose, even without destroying neurons (Shimura et al., 2006; Ho and Berridge, 2014).

Recently, to more accurately map the site in VP responsible for production of disgust, Chao-Yi Ho compared the effects of small excitotoxic lesions to localized pharmacological inactivation in LH, VP, and extended amygdala (Ho and Berridge, 2014). Ho and Berridge found that the posterior half of VP was the primary site for inducing “disgust”, both by temporary muscimol inactivations and by excitotoxic lesions. Posterior VP sites of lesions/inactivations were more effective at inducing “disgust” reactions than anterior VP sites, or than any sites outside VP, such as in LH, LPO or extended amygdala. Ho and Berridge’s findings suggest that the posterior subregion of VP is especially important for normal hedonic impact, because posterior VP is the only site in the brain known so far in which lesions or inactivations replace positive hedonic reactions to sweetness with aversive “disgust” reactions.

## Ventral Pallidum Hedonic Hotspot

**Figure 1:**
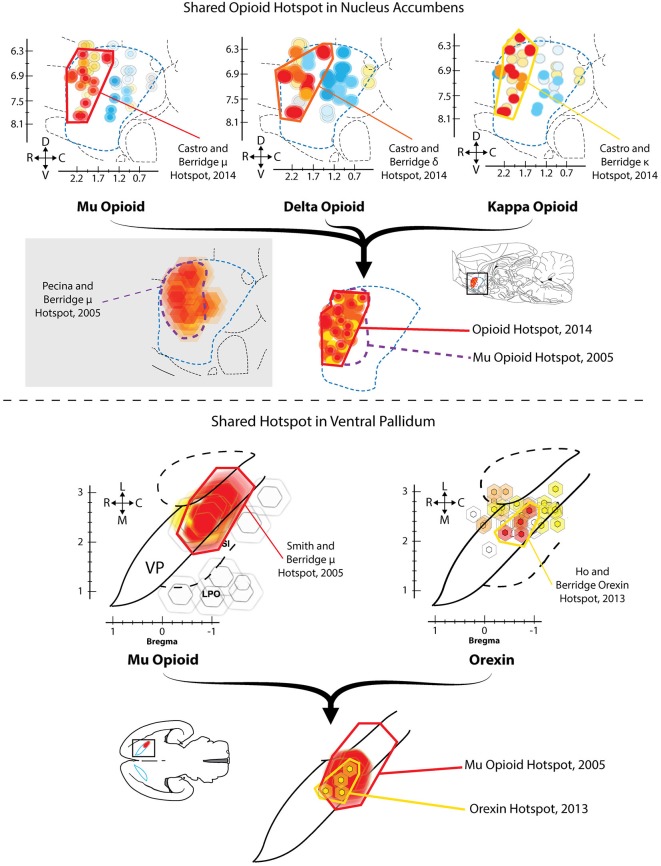
**Hedonic hotspots in nucleus accumbens (NAc) and ventral pallidum (VP)**. *Top*: Sagittal views of NAc medial shell showing functional maps of opioid-stimulated hedonic enhancements, as measured by taste reactivity to sucrose. Microinjections that stimulated mu (left), delta (middle) or kappa (right) opioid receptors in the rostrodorsal quadrant of NAc medial shell increased hedonic “liking” reactions to sucrose, compared to vehicle baselines in the same rats. A single map showing shared opioid hotspot includes any site where agonist microinjection increased hedonic reactions by at least 250% compared to baseline (overlapping sites shown adjacently). Mu effects are depicted in red, delta in orange, and kappa in yellow (Modified from Castro and Berridge, 2014). *Bottom*: Horizontal maps of VP similarly show sites where mu opioid (left) or orexin stimulation increased hedonic reactions. A shared hedonic hotspot is revealed in posterior VP, where either mu opioid or orexin stimulation increased hedonic reactions by at least 150% compared to vehicle baseline in the same rats. Mu is colored red, and orexin is colored yellow (Modified from Ho and Berridge, 2013).

Given the apparent importance of VP for generating normal hedonic impact, a related question is: does any region of VP also increase or boost the hedonic impact of a taste? In a first demonstration that VP contains a “hedonic hotspot”, namely, a site capable of increasing the number of positive “liking” reactions elicited by sweetness, Smith and Berridge (2005) performed a microinjection mapping study of VP using the mu opioid receptor agonist DAMGO. Smith found that mu opioid receptor stimulation specifically at sites in the caudal half of VP caused robust increases in the number of hedonic “liking” reactions elicited by sucrose taste, as well as increases in food intake (Figure 1). This site was roughly 0.8 mm^3^ in volume, and localized to the posterior half of VP (and the same site as described above where neuronal damage/inhibition causes “disgust”). By contrast, the same opioid stimulation in the rostral half of VP suppressed “liking” reactions to sucrose (i.e., a hedonic coldspot), and suppressed food intake.

Lastly, and in contrast to the hedonic effects of mu opioid receptor stimulation, microinjections of the GABA antagonist bicuculline stimulated food intake throughout the entire VP, but never altered taste reactivity orofacial patterns at any VP site (Smith and Berridge, 2005).

## Ventral Pallidum Orexin Hotspot

Ho and Berridge recently confirmed that the posterior VP contained an opioid-mediated hedonic hotspot, and additionally found that orexin stimulation in the same caudal VP site, via microinjections of orexin-A, similarly increased hedonic “liking” reactions to sucrose taste (Ho and Berridge, 2013). Orexin (also known as hypocretin) is a peptide implicated in hunger and arousal that is only produced in hypothalamus (Peyron et al., 1998; Baldo et al., 2003; Swanson et al., 2005). A hypothalamic subregion localized to the perifornical and mid-tuberal region of LH contains orexin neurons that appear especially important for food and drug reward (Harris et al., 2005; Aston-Jones et al., 2010). Orexin neurons also extend medially to dorsomedial hypothalamus and other hypothalamic subregions, where orexin influences arousal, sleep/wake cycles and attention (Espana et al., 2001; Adamantidis et al., 2010). LH orexin neurons project to numerous sites throughout the brain, reaching targets as far caudal as the brainstem parabrachial nucleus of the pons, and as far rostral as orbitofrontal cortex (Peyron et al., 1998; Baldo et al., 2003). Importantly, orexin neurons in LH also appear to project to VP (Peyron et al., 1998; Baldo et al., 2003), and immunoreactivity for orexin receptors has been found in caudal VP (Marcus et al., 2001), suggesting connectivity and receptor mechanisms for functional hedonic effects of orexin in VP.

Ho and Berridge compared the effects of orexin microinjections at sites distributed in VP and in anterior LH, and found that only at sites in caudal VP did orexin increase hedonic “liking” reactions (Figure 1; Ho and Berridge, 2013). In that posterior VP hotspot, orexin stimulation selectively increased positive “liking” reactions to sucrose, without altering negative “disgust” reactions elicited by quinine. Orexin microinjections in nearby LH or extended amygdala failed to alter orofacial responses to either sucrose or quinine. These results collectively show that the hotspot of posterior VP can use either orexin or opioid signals to similarly increase the positive hedonic impact of a sweet taste.

More recently, pilot studies in our lab have used optogenetic techniques to further explore VP and LH roles in hedonic “liking” reactions to sucrose. In preliminary optogenetic studies, we infused a channelrhodopsin-carrying virus into LH neurons in the reward-relevant perifornical-midtuberal site of orexin neurons, and subsequently stimulated LH projection terminals via an optic fiber placed within the posterior VP hotspot (Castro and Berridge, 2013). Our preliminary results suggest that optogenetic stimulation of infected LH terminals within the VP does increase hedonic reactions to sucrose without altering “disgust” reactions to quinine, and also increases food intake. In addition, some rats were infused with virus into the posterior VP itself along with implantation of an optic fiber in the VP hotspot, or alternatively, infused with virus and implanted with optic fibers both in the LH. Our results show that optogenetic stimulation of intrinsic VP neurons (cell bodies) similarly cause an increase of “liking” reactions to sucrose taste. By contrast, stimulation of intrinsic LH neurons only caused an increase in food intake without enhancing “liking” reactions to sweetness (Castro and Berridge, 2013). That is, intrinsic LH neurons appear to predominantly influence food intake or “wanting” to eat, whereas VP hotspot neurons or specific LH projections to VP more effectively enhance “liking”. This pattern creates the puzzle of why stimulation of LH axon terminals in posterior VP, but not direct stimulation of LH cell bodies at LH sites, increased affective reactions to sucrose. One possible reason is that intrinsic LH stimulation broadly activates neurons that project to many other brain sites besides the VP, and some of those additional projections may have effects that oppose or compete with the hedonic effects of selective LH-to-VP hotspot stimulation. While this possibility needs further confirmation, these preliminary results support previous suggestions that LH and VP mediate separable “wanting” vs. “liking” aspects of food reward.

## Nucleus Accumbens Hotspot

Similar to the VP, the NAc also contains an opioid-mediated hedonic hotspot where opioid stimulation can increase “liking” reactions to sucrose taste. The NAc hotspot is approximately one cubic millimeter in volume, and is located in the rostrodorsal quadrant of medial shell (Peciña and Berridge, 2005; Castro and Berridge, 2014). This NAc opioid hotspot was discovered using a similar microinjection and functional mapping technique of effects on taste reactivity behaviors that was used for the VP hotspot. Microinjections of the mu agonist DAMGO were compared at many sites throughout the medial shell of NAc. Results showed that only stimulation within the rostrodorsal quadrant of NAc medial shell caused a three-fold increase of “liking” reactions to sucrose (Peciña and Berridge, 2005). By contrast, mu receptor stimulation in a similarly-sized hedonic coldspot located in the caudal half of NAc medial shell actually reduced hedonic reactions to sucrose. At all other locations in medial shell, DAMGO microinjections induced no hedonic change in taste reactivity to sucrose. However, DAMGO microinjections at all sites located anywhere in NAc medial shell produced increases in food intake two-times to eight-times higher than baseline intake, even at caudal sites (Peciña and Berridge, 2005). This pattern demonstrated an anatomical distinction between NAc mu opioid stimulation of “liking” (i.e., specific to the rostral hotspot) vs. NAc mu stimulation of “wanting” for food (i.e., anywhere in NAc medial shell). The stimulation of “wanting” also confirmed previous reports that DAMGO microinjections in NAc increased food intake at virtually all sites in the NAc, including both medial shell and core (Zhang and Kelley, 2000). Thus, opioid neurocircuitry for enhancing motivated “wanting” to consume food rewards is more widely distributed in NAc than opioid circuitry for enhancing hedonic “liking” in the same structure. Indeed, opioid mechanisms for “wanting” without “liking” extend to several other structures, including regions of amygdala, neostriatum, and prefrontal cortex (Zhang and Kelley, 2000; Mahler and Berridge, 2009; Mena et al., 2011; Difeliceantonio et al., 2012).

Recently, we have replicated the localization of the opioid hedonic hotspot in NAc, showing that mu opioid stimulated increases of hedonic reactions to sucrose are limited to DAMGO microinjection sites in the rostrodorsal quadrant of medial shell, whereas mu opioid stimulation anywhere in NAc medial shell increases food intake (Castro and Berridge, 2014). In this recent study, we also found that stimulation of delta opioid receptors and even kappa opioid receptors could increase sucrose hedonic impact, but only within the same rostrodorsal hotspot of medial shell. At other sites in the coldspot of the caudal half of medial shell, all kappa, delta, and mu opioid stimulations suppressed “liking” reactions to sucrose, confirming the localization of the NAc caudal coldspot as well as rostral hotspot (Figure 1; Castro and Berridge, 2014). Delta stimulation in the rostral hotspot, but not at other NAc shell locations, also increased food intake, whereas kappa stimulation at any site in medial shell never consistently increased food intake, despite enhancing “liking” reactions within the rostral hotspot. These findings indicated a surprising degree of localization for hedonic functions shared by all three major types of opioid receptors, and indicated that the NAc mechanisms mediating “liking” vs. “wanting” for a food reward can be distinguished even within a particular type of opioid receptor.

## Nucleus Accumbens Rostrocaudal Gradient

Anatomical localization of function applies to motivation-generating mechanisms as well as hedonic-enhancing mechanisms in NAc medial shell. Manipulations of amino acid neurotransmission in particular locations of NAc medial shell can produce localized induction of intense affective and motivated states that have opposite valence, such as desire (i.e., positively valenced in the sense that microinjections of either a GABA-A agonist or glutamate AMPA antagonist produce positive place preference and appetitive eating behavior) vs. dread (i.e., negatively valenced in the sense that microinjections produce negative place avoidance, fearful vocalization, biting and defensive treading) (Reynolds and Berridge, 2001, 2008; Richard et al., 2013). Of course, “desire” and “dread” are only shorthand terms for the motivated states elicited by amino acid modulating drugs microinjections in NAc shell, but are useful for referring to the different behavioral patterns and localization of function observed after these NAc manipulations. One possible psychological explanation has been suggested to involve induction of intense motivational salience that becomes attributed to particular sensory percepts. These percepts can be either positively-valenced as incentive salience or negatively-valenced as fearful salience (i.e., the sight of food a pellet becomes more salient and attractive after rostral shell microinjections; the sight of light reflecting off glittering surfaces or of objects in the room beyond becomes more attention-grabbing, but is perceived as threatening after caudal shell microinjections). A possible neurobiological mechanism involved may be that a GABA agonist or glutamate antagonist microinjection induces a relative inhibition of GABAergic medium spiny neurons (MSNs) within NAc shell. This could then disinhibit distinct downstream projections to targets such as LH, VP, or VTA from the tonic suppression that is usually exerted by NAc GABAergic projections, releasing those target sites to actively generate the motivated behaviors (Mogenson et al., 1983; Zahm and Heimer, 1990; Heimer et al., 1991; Lu et al., 1998; Usuda et al., 1998; Zhou et al., 2003; Humphries and Prescott, 2010).

An anatomical localization of NAc function applies to the production of these motivated behaviors, possibly related to the rostral hotspot and caudal coldspot described above. That is, GABA/glutamatergic manipulations at rostral sites in NAc medial shell typically produce the appetitive behaviors, whereas the same neurochemical manipulations at caudal sites in NAc shell instead produce the fearful or defensive behaviors. Sites in the middle of NAc can often elicit a mixture of appetitive and fearful behaviors from the same rat during the same 1h test. Thus, studies in our lab have found these intense motivations to be generated along a rostrocaudal “keyboard-type” pattern induced by localized disruptions via microinjections of DNQX or muscimol (Reynolds and Berridge, 2001, 2002, 2003, 2008; Faure et al., 2008; Richard and Berridge, 2011, 2013). Although there are only two valences of motivation evoked (i.e., positive desire vs. negative dread), progressive shifts along the rostrocaudal axis produces many different quantitative mixtures of appetitive/defensive responses. In that sense, the multiple, diverse outputs are analogous to the diverse musical notes (varying continuously along a frequency gradient) produced by moving a brick along a musical keyboard: the sounds change progressively in pitch as the brick moves in one direction, just as elicited mixtures of desire/dread behaviors change progressively in valence as NAc microinjection sites are moved along the anterior-posterior gradient of medial shell. Alternatively, the rostrocaudal gradient could be compared to a color spectrum in which single a continuous shift from 470 nm (perceived as blue) to 530 nm (perceived as green) produces distinct mixtures of blue–green hues that appear qualitatively different from each other.

In particular, as sites move from the anterior end of shell in a posterior direction, the intensity of appetitive behaviors gradually declines while the intensity of fearful behaviors simultaneously and incrementally increases (Reynolds and Berridge, 2001, 2002, 2003, 2008). Additionally, GABAergic muscimol microinjections also recruit hedonic reactions of “liking” vs. “disgust” reactions to tastes along this same axis: positive “liking” at rostral sites vs. “disgust” at caudal sites in medial shell.

Others have shown that GABA-A stimulation of food intake in rostral shell sites, requires VP and LH recruitment, as pharmacological inhibition or lesion of VP or LH attenuates the NAc-induced increase in eating (Stratford and Kelley, 1999; Stratford and Wirtshafter, 2012; Urstadt et al., 2013a,b). Downstream recruitment of structures by caudal shell sites for generating fearful reactions has not yet been explored, although the D1/D2 pattern of dopamine receptor dependence discussed below is consistent with recruitment of VP and LH.

However, it could be questioned whether increases in eating are truly appetitive when evoked by microinjections of GABA agonist or glutamate antagonist in NAc shell, in the sense of being mediated by incentive salience or “wanting”. Alternatively, increased food intake could be viewed as pure motor activity, or as due to an aversive drive (Solomon and Corbit, 1974; Koob, 1996).

To help decide, we note that incentive salience has signature features, when attributed to unconditioned reward stimuli such as food, or to related Pavlovian conditioned stimuli or cues (Robinson and Berridge, 1993). Those features are evident after many brain manipulations that increase incentive salience, such as dopamine or opioid stimulations in NAc, amygdala or neostriatum (Wyvell and Berridge, 2000; Mahler and Berridge, 2009; Smith et al., 2011; Difeliceantonio et al., 2012; Peciña and Berridge, 2013). In brief, a Pavlovian conditioned stimulus (CS+) for reward is considered have incentive salience if it meets the following conditions: (1) it is attractive or acts as a “motivational magnet” (e.g., elicits approach such as sign-tracking or goal-tracking) (DiFeliceantonio and Berridge, 2012; Robinson and Berridge, 2013; Yager and Robinson, 2013; Yager et al., 2014); (2) the CS+ becomes “wanted” itself, in the sense an individual will work for it (typically measured in instrumental conditioned reinforcement tests as operant responding for CS+ alone); and (3) the CS+ spurs pulses of higher motivation to obtain its unconditioned reward (typically measured in Pavlovian-Instrumental Transfer (PIT) tests, or in priming of consumption tests). Yet, while GABA agonist and glutamate antagonist microinjections in NAc shell powerfully increase motivated behaviors toward unconditioned stimuli (e.g., UCSs such as the sight and smell of chow pellet; sight and touch of approaching human hand; sight of glittering light or external movement), those amino acid manipulations often fail to enhance learned appetitive motivation toward Pavlovian cues. For example, Zhang et al. (2003) and Hanlon et al. (2004) reported that muscimol microinjections into the NAc shell failed to increase instrumental acquisition or breakpoint effort to earn food on a lever pressing task (Zhang et al., 2003; Hanlon et al., 2004). Similarly, muscimol microinjections in NAc fail to increase cue-triggered “wanting” on a PIT task (Corbit and Balleine, 2011). GABA/glutamate failures can be contrasted to opioid or dopamine manipulations in NAc shell, both of which positively enhance learned appetitive motivations involving CS+s. One reason why opioid or dopamine stimulation in NAc may be better able to enhance learned appetitive performance is that opioid/dopamine signals act as neuromodulators. As modulators, they can subtly alter complex endogenous signals that convey information about learned external stimuli and associated representations. By comparison, GABA and glutamate amino acid neurotransmitters may act as the signals themselves, significantly hyperpolarizing or depolarizing NAc neurons. Therefore, drugs that act on GABA or glutamate receptors may actually disrupt endogenous signals (i.e., by either preventing or mimicking those signals), rather than amplifying endogenous signals, as opioid or dopamine agonists may. Learned Pavlovian cues may be relatively vulnerable to signal disruption, because learning may recruit highly complex patterns of signals in brain circuits, whereas unconditioned stimuli (i.e., the sight and smell of actual food) may be more robust, and so resist disruption after NAc GABA or glutamate microinjections. This may be one reason why muscimol and DNQX microinjections can increase appetitive/defensive behavior elicited by unconditioned stimuli, yet not so reliably increase related motivated behaviors elicited by learned cues.

Still, the CS/UCS difference is not absolutely categorical: there are other reports that muscimol or DNQX microinjections in NAc can sometimes succeed in enhancing learned behaviors for food reward, as well as increasing unconditioned consumption of food itself. For example, Wirtshafter and Stratford reported that muscimol microinjections in NAc enhance responding for sucrose reward on an FR1 instrumental schedule (Wirtshafter and Stratford, 2010; Stratford and Wirtshafter, 2012), similar to amphetamine microinjections. Furthermore, muscimol or DNQX microinjections in rostral NAc sites have been shown to establish appetitive conditioned place preferences (CPPs) for an associated location (Reynolds and Berridge, 2002, 2003), similar to dopamine and opioid agonists (Liao et al., 2000; Castro and Berridge, 2014). Conversely, DNQX and muscimol microinjections into caudal NAc sites have been found to establish conditioned place avoidances (Reynolds and Berridge, 2002, 2003). Thus, while amino acid transmitter manipulations in NAc do not necessarily bear all the signature features of incentive salience, there are reasons to conclude that their incentive motivation effects overlap with some features of “wanting”.

## Amino Acids Differ for Hedonic Impact in Nucleus Accumbens

While glutamate and GABA manipulations in NAc produce similar patterns of motivated desire (eating) or dread (defensive treading, escapes, etc.), the two manipulations differ strongly regarding effects on hedonic impact or “liking”. GABA inhibition of neurons in a rostral strip of medial shell increases hedonic “liking” reactions to a bittersweet sucrose/quinine solution, in addition to stimulating food intake and establishing a CPP (Reynolds and Berridge, 2002; Faure et al., 2010). By contrast, GABAergic agonist microinjections at sites in the caudal half of shell decrease “liking” reactions and increase negative “disgust” reactions. Unlike GABA agonism, blockade of glutamate AMPA receptors by DNQX microinjections in NAc shell fail to amplify positive “liking” reactions at rostral sites, and fail to produce “disgust” reactions at caudal sites, even though they produce equal degrees of desire or dread behaviors as muscimol at those same sites (Faure et al., 2010). These findings indicate that glutamate-mediated appetitive/fearful effects are restricted to only motivation states without corresponding effects on hedonic impact, whereas the GABA-mediated induction of appetitive/fearful motivation states likewise affects “liking” or “disgust”.

## Environmental Retuning of Glutamatergic NAc Keyboard

**Figure 2:**
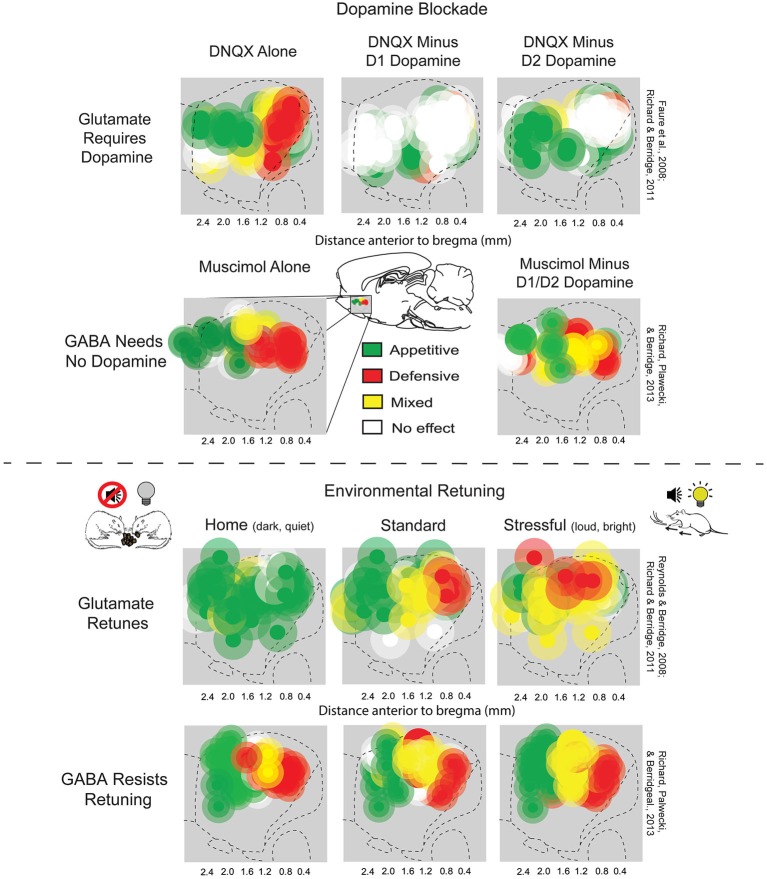
**NAc rostrocaudal differences in glutamate and GABA mediation of desire and dread**. *Top*: Sagittal view showing maps of DNQX- or muscimol-generated appetitive eating vs. fearful or defensive behaviors, and effects of adding local dopamine blockade to the same microinjection. Appetitive eating and defensive reactions elicited by DNQX (top left) both require D1 receptor function (top middle), but defensive reactions additionally require D2 receptor function (top right) (figure is adapted from Richard and Berridge, 2011). By contrast, GABAergic muscimol-generated eating and defensive behaviors (bottom left) are unaffected by dopamine blockade (bottom right) (adapted from Richard et al., 2013). *Bottom*: Environmental ambience retunes the motivation function of NAc sites. Testing in home environments abolished almost all defensive reactions, and promoted appetitive eating, elicited by DNQX microinjections in NAc shell (top left). By contrast, a stressfully loud and bright environment expanded DNQX-generated defensive behaviors into rostral regions of NAc shell, and suppressed fearful reactions (top right), relative to standard laboratory conditions (top middle). Muscimol microinjections in NAc shell generated eating and fear at similar sites to DNQX, but was not shifted by environmental ambience of home (bottom left), standard (bottom middle), or stressful test conditions (bottom right). Sites are colored as producing appetitive behavior (green sites), defensive behavior (red sites), or mixtures of both behaviors (yellow). Green symbols represent at least a 200% increase in food intake, red symbols represent at least a 500% increase in defensive treading or another defensive reaction (distress vocalization, escape attempt or biting when rat was touched), and yellow symbols represent a combination of the eating and defensive criteria (adapted from Richard et al., 2013).

Another way in which muscimol and DNQX effects in NAc medial shell differ from each other is that DNQX-induced motivations are susceptible to retuning by shifts in environmental ambience (Figure 2; Reynolds and Berridge, 2008). By contrast, GABA-induced motivations resist environmental retuning and are more permanently fixed in valence by anatomical site (Richard et al., 2013). For example, when rats were placed in a dark and quiet home-cage environment, which they prefer over standard laboratory conditions, zones in which DNQX elicited appetitive food intake expanded from rostral shell to also include caudal shell sites, while fear-generating zones shrunk to a small far-caudal strip, and remaining fear reactions were reduced. Conversely, when rats were given a DNQX microinjection and placed in a stressful, over-stimulating environment with bright lights and loud rock music (which rats avoid if given a choice), caudal fear-inducing zones expanded to the rostral half of medial shell, while appetitive, desire-generating zones shrunk to a small far-rostral strip. Thus, the same DNQX microinjection, in the same NAc site, and in the same rat, can elicit oppositely-valenced motivated behaviors depending on ambient conditions (Reynolds and Berridge, 2008; Richard and Berridge, 2011; Richard et al., 2013).

The environmental modulation of motivation may involve signals from amygdala, cortex, and related sources that project to NAc. Gill and Grace (2011) demonstrated that stimulation of basolateral amygdala produces decreases in rostral NAc firing rates and increases in caudal NAc firing rates, and suggested such changes might contribute to environmental retuning of desire-dread evoked from NAc shell. Ventral hippocampus stimulation also produced differences in recorded firing rates between rostral and caudal NAc shell. Additionally, inactivation of ventral hippocampus via tetrodotoxin abolished basolateral amygdala-induced changes in NAc activity, indicating that interactions between NAc, vHipp, and amygdala might influence NAc-related motivations. Finally, corticolimbic projections from prefrontal cortex to NAc may play a role in retuning NAc-mediated generation of appetitive/fearful motivations. In support of this, Richard et al. (2013) found that disinhibition (excitation) of infralimbic cortex decreased both appetitive and fearful motivations induced by NAc shell microinjections of DNQX. By comparison, disinhibition of medial orbitofrontal cortex enhanced NAc DNQX induction of appetitive behaviors, at least in the otherwise fear-producing zone of caudal shell. Collectively, these findings implicate amygdala, hippocampus, and prefrontal cortex projections to NAc in environmental retuning of NAc production of appetitive/fearful motivation.

## NAc Dopamine Roles in Glutamatergic Desire and Dread

Neurochemically, glutamate and dopamine signals also interact on NAc neurons (Sesack and Pickel, 1992; Hanlon et al., 2004; Surmeier et al., 2007; Carlezon and Thomas, 2009; Bromberg-Martin et al., 2010; Lammel et al., 2014). Indeed, endogenous dopamine is required for the glutamatergic “keyboard” generation of motivated behaviors by NAc DNQX microinjections. Alexis Faure and Jocelyn Richard in our lab first found that adding a combined D1 antagonist plus D2 antagonist to the same microinjection which contained DNQX completely blocked the generation of eating or fearful reactions by the glutamatergic antagonist, at all sites in medial shell (Faure et al., 2008). Subsequently, Richard and Berridge examined the separate roles of D1 blockade vs. D2 blockade, and found that DNQX-generation of appetitive food intake was prevented only by the blockade of local D1 dopamine receptors, and never reduced by local D2 blockade (Figure 2; Richard and Berridge, 2011). In contrast, local D2 blockade, in addition to local D1 blockade, completely prevented the DNQX generation of fearful behaviors, showing that endogenous activity was required simultaneously at both D1 and D2 dopamine receptors for inducing the intense fearful reactions (Faure et al., 2008; Richard and Berridge, 2011). This D1 vs. D2 pattern even held true for individual middle NAc sites along the anterior-posterior axis when their valence was retuned by shifts in homelike vs. stressful environmental ambience. That is, in the comfortable environment of the rat’s home cage, appetitive eating produced by DNQX did not require D2 dopamine activation, but did require D1 activation. Conversely, in a stressfully bright and loud environment, fearful motivation generated at the same site did require D2 receptor activation, as well as D1 activation (Richard and Berridge, 2011). By contrast to dopamine dependence for glutamatergic signaling, GABAergic generation of motivations by muscimol microinjections in NAc shell never required either D1 or D2 type receptors (Figure 2; Richard et al., 2013).

## Implications for NAc Direct vs. Indirect Paths

The difference between D1 and D2 dopamine requirements for NAc glutamate-mediated generation of appetitive vs. fearful motivations possibly reflects differential recruitment of parallel output pathways from medial shell. A “direct” projection pathway from NAc to the VTA comprises NAc neurons that express only D1 receptors (though those D1 neurons may also project to LH and VP, in a dilution of the stricter direct/indirect dichotomy found dorsally in neostriatum) (Heimer et al., 1991; Lu et al., 1998; Zhou et al., 2003; Humphries and Prescott, 2010). By contrast, NAc neurons that express D2 receptors all belong to the indirect path that projects only to LH and VP (adhering to the indirect rule of projecting to intermediary forebrain targets before relaying indirectly to midbrain), and never directly from NAc to VTA. Additionally, approximately 35% of MSNs co-express both D1 and D2 receptors, and are thought to belong to the indirect path (Bertran-Gonzalez et al., 2008; Humphries and Prescott, 2010; Perreault et al., 2010, 2011). Thus, both D1 and D2 expressing NAc neurons project indirectly to VTA via VP and LH, but only D1 neurons project directly to midbrain VTA. In light of this pattern, the requirement for both D1 and D2 endogenous signals in fear induced by NAc microinjections of DNQX may imply that both the direct pathway and indirect pathway are recruited in generating fearful states. Conversely, the sole dependence on D1 signals, but not D2 signals, in DNQX-induced eating could reflect a predominant contribution of the D1 direct pathway neurons that project to VTA in generating appetitive states (or of specific D1 NAc projection to indirect LH and VP targets, different from D2 NAc projections).

## Inhibition vs. Excitation of Down Stream Targets

What NAc neurobiological events produce the intense motivations described above? Specifically, what are the relative roles of NAc neuronal inhibition vs. NAc neuronal excitation? One popular hypothesis is that the inhibitory hyperpolarization of MSNs in NAc is the primary mechanism for generating appetitive motivation (Carlezon and Wise, 1996; Cheer et al., 2005; Roitman et al., 2005, 2008; Taha and Fields, 2006; Meredith et al., 2008; Wheeler et al., 2008; Carlezon and Thomas, 2009; Krause et al., 2010). The inhibition of NAc projection neurons is viewed by this hyperpolarization hypothesis to release downstream neurons in target structures from chronic GABAergic suppression, and consequently disinhibit those target neurons into states of excitation. This hypothesis is supported by findings that neural excitations in downstream targets, such as VP, LH, or VTA occur during reward events (Ljungberg et al., 1991; Baldo et al., 2004; Stratford, 2005; Bromberg-Martin and Hikosaka, 2009; Tindell et al., 2009; Smith et al., 2011). Furthermore, the NAc inhibition hypothesis fits the desire-dread “keyboard” effects of inhibitory drug microinjections, such as muscimol (a GABA agonist which should hyperpolarize NAc neurons) or DNQX (a glutamate AMPA antagonist which should induce relative NAc inhibition by preventing glutamatergic depolarization). It also has been suggested to apply to opioid agonists, on the presumption that those drugs have generally inhibitory effects (Kelley et al., 2005; Baldo and Kelley, 2007; Carlezon and Thomas, 2009).

Further support for the hyperpolarization hypothesis comes from electrophysiological reports that NAc neurons are most likely to show inhibitions of firing evoked by drug or sweet rewards (Peoples and West, 1996; Chang et al., 1997; Janak et al., 1999; Nicola et al., 2004a; Roitman et al., 2005, 2010). Conversely, aversive tastes of bitter quinine evoke excitatory increases in firing (Roitman et al., 2005). Additionally, NAc neurons switch from reductions in firing to increases in response to a sweet taste that has become disgusting following acquisition of a Pavlovian taste aversion, and neuronal inhibition to the taste of food is augmented by physiological hunger that makes the taste more rewarding (Hollander et al., 2002; Wheeler et al., 2008; Roitman et al., 2010). Similarly, physiological states of salt depletion cause the normally aversive taste of hypertonic NaCl to become palatable, switching NAc neuronal responses from excitation to inhibition. Furthermore, thirst states are also seen to augment the inhibition of firing to the taste of water (Hollander et al., 2002; Loriaux et al., 2011).

Yet, beyond this evidence for NAc neuronal inhibition in reward, other evidence exists that rather confusingly points toward an opposite conclusion: NAc neuronal *excitation* also may mediate motivation and reward. For example, electrophysiological studies by Roitman et al. (2005, 2010) and Wheeler et al. (2008) reported that approximately 30% of NAc core and shell neurons increased in firing in response to sweet rewards (Roitman et al., 2005, 2010; Wheeler et al., 2008). Taha and Fields (2005) reported that nearly 75% of shell and core neurons in NAc showed increases in firing elicited by sucrose rewards, with highest firing to the most concentrated sucrose solution. Additionally, several other electrophysiological studies report that approximately 30%–50% of NAc shell and core neurons increase firing during anticipation or during instrumental actions aimed at obtaining food, water or cocaine rewards (Carelli, 2000; Carelli et al., 2000; Hollander et al., 2002; Nicola et al., 2004b).

A second line of evidence for NAc excitation in reward comes from several decades of studies on NAc electrode self-stimulation in rats. That is, rats will work to activate depolarizing electrodes in NAc sites, implying that excitation of some NAc neurons is sufficient as a reward (Rolls, 1971; Phillips and Fibiger, 1978; Mogenson et al., 1979; Van Ree and Otte, 1980; Phillips, 1984). Similarly, human deep brain self-stimulation has been reported for patients who have had electrode sites that likely included NAc (Heath, 1972, 1996). However, the exact effects of electrodes on nearby neurons is admittedly complex, and has been suggested to involve neuronal disruption as well as neuronal stimulation (Ranck, 1975).

Contemporary optogenetic techniques allow for more specific stimulation of particular neurons, and can better ensure that neuronal depolarization is the neurobiological mechanism of an observed behavioral effect. Recent optogenetic studies have shown that direct excitatory depolarization of neurons in NAc, via laser activation of channelrhodopsin-2 photoreceptors (ChR2) supports self-stimulation (Britt et al., 2012). Furthermore, ChR2 stimulation of NAc shell neurons has also been shown to potentiate a cocaine-induced CPP, suggesting that depolarization of NAc neurons can also enhance drug reward (Lobo et al., 2010).

We have recently used ChR2 photoexcitation to explore the role of NAc neuronal excitation in reward, selectively targeting either the D1 receptor-expressing subpopulation of NAc neurons in medial shell (i.e., including those that belong to direct path) or D2 subpopulation of NAc neurons (i.e., including only those that belong to the indirect path) (Cole et al., 2014). NAc neurons expressing D1 receptors can be selectively activated in transgenic mice that express Cre recombinase only in D1 neurons, by microinjecting into NAc a ChR2 virus in tandem with a Cre-dependent, double-flox inverted (DIO) construct (Gong et al., 2007; Sohal et al., 2009; Kravitz et al., 2010, 2012; Lobo et al., 2010). The result is to selectively express ChR2 photoreceptors within Cre-positive D1 neurons of NAc, but not in other NAc neurons. Conversely, D2 neurons can be selectively activated by using different transgenic mice that express Cre recombinase only in their D2-expressing neuron, with the same Cre-dependent ChR2 virus and optic fiber placement in the NAc. Although a subpopulation of approximately 36% of neurons in NAc does express both types of dopamine receptors, this approach still predominantly targets two mostly different neuronal populations. And if D1 stimulation vs. D2 stimulation produces different behavioral effects, this approach can reveal which of the two NAc neuronal populations contributes most to motivation for reward.

In our lab, D1 Cre and D2 Cre strains of mice have been tested for self-stimulations by contacting a particular designated metal object (an empty drinking spout): any touch with their nose, mouth or paws delivers a brief laser illumination to NAc shell, activating their particular NAc neuronal subpopulation. A second metal spout was also available, but delivered no laser stimulation, and served merely as a control stimulus to detect any general tendency to touch objects via exploration or general activity (which could be subtracted from touch counts for the self-stimulation spout). Our preliminary results suggest that specific excitation of D1 MSNs in NAc shell of D1-Cre mice produces robust self-stimulation behavior (Cole et al., 2014). That seems similar to D1 self-stimulation reported for dorsal regions of neostriatum (Kravitz et al., 2012). Our finding of NAc D1-neuronal self-stimulation also seems consistent with reports of D1-enhanced CPP established by morphine or cocaine (Lobo et al., 2010; Koo et al., 2014). By contrast, our preliminary results suggest that specific stimulation of D2 neurons in NAc fails to produce self-stimulation when selectively excited (Cole et al., 2014). Interestingly, NAc D2 stimulated mice also do not show any detectable avoidance of laser stimulation in our hands, which contrasts with avoidance of D2 stimulation in neostriatum sites reported by others (Kravitz et al., 2012). The neutral response to NAc D2 stimulation also seems to conflict with expectations from reports that D2 stimulation in NAc attenuates drug reward (Lobo et al., 2010; Koo et al., 2014). These issues will need to be further examined in future studies, but it appears that excitation of NAc D1 neurons mediates appetitive motivation for reward, whereas excitation of D2 neurons does not.

Beyond direct excitation of intrinsic neurons of NAc, a final line of support for NAc excitation in reward is evidence that there are reward effects of stimulating excitatory glutamatergic inputs to NAc, especially from prefrontal cortex, (Britt et al., 2012) basolateral amygdala, and hippocampus (Will et al., 2004; Ambroggi et al., 2008; Britt et al., 2012). For example, Ambroggi et al. (2008) reported that glutamatergic inputs from the BLA to NAc were required for cue-triggered seeking of sucrose reward. Others have reported that optogenetic excitation of glutamatergic projections from prefrontal cortex, BLA, or ventral hippocampus to NAc produces self-stimulation or CPP effects (Stuber et al., 2011; Britt et al., 2012). These observations suggest that glutamate release from those structures excites NAc neurons to contribute to reward processes.

How can one reconcile the NAc hyperpolarization hypothesis of reward with the NAc depolarization hypothesis? That remains a puzzle to be resolved in the future, but one possibility is that different NAc neuronal subpopulations mediate hyperpolarization vs. depolarization generation of reward motivation (Carelli et al., 2000; Nicola et al., 2004a, b; Roitman et al., 2005; Taha and Fields, 2005; Ghazizadeh et al., 2012). It is also possible that direct depolarization of some NAc MSNs may subsequently facilitate hyperpolarization of other NAc neurons via local GABAergic projections or interneurons that produce lateral inhibition. Alternatively, perhaps some NAc neurons have a number of distinct polarization modes of function to produce motivation or reward. For example, Sun and Laviolette (2014) reported that excitation of medium spiny neurons and inhibition of fast-spiking interneurons in NAc produced positive reward effects, whereas inhibition of medium spiny neurons and excitation of fast-spiking interneurons produced negative avoidance effects in a place preference/avoidance task. Additionally, as Kravitz and Bonci (2013) have suggested, the temporal pattern of neuronal firing may also prove relevant in determining behavioral effects. Future work will be needed to clarify the relative roles of NAc neuronal inhibition vs. excitation in reward and motivation.

## Synthesis of NAc “D1-Direct” vs. “D2-Indirect” Microinjection and Optogenetic Studies

**Figure 3:**
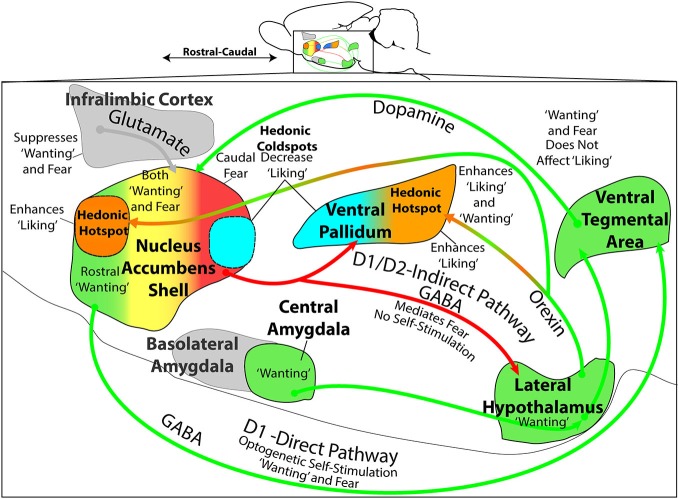
**Mesocorticolimbic-hypothalamic circuitry and functions**. Sagittal view depicts structures and circuitry underlying “liking”, “wanting”, and “fear” functions discussed in text. The NAc medial shell contains a hedonic hotspot in the rostral half, where opioid and related stimulation increases “liking” reactions to sucrose taste. Conversely, NAc shell contains a caudal hedonic coldspot, where opioid stimulation suppresses “liking” reactions to sweetness. These functional sites overlap with the NAc shell motivational keyboard for GABAergic/glutamatergic microinjections, in which rostral sites produce desire (e.g., eating, place preference, etc.), caudal sites produce fear (e.g., antipredator reactions, distress calls and bites, and place avoidance). Furthermore, NAc glutamatergic generation of appetitive behavior by DNQX microinjection requires endogenous dopamine stimulation of D1 receptors on direct path neurons that project directly to ventral tegmental area (VTA). Optogenetic stimulation of NAc D1-expressing neurons also supports appetitive self-stimulation behavior throughout the entire medial shell. By contrast, NAc glutamatergic generation of fearful behaviors requires additional dopamine stimulation of D2-receptors, implicating indirect path neurons that project to the VP and lateral hypothalamus (LH). The posterior half of VP contains an opioid hedonic hotspot, whereas the rostral half of VP contains a coldspot where mu opioid stimulation suppresses “liking” reactions to sucrose taste. Optogenetic stimulation of the VP hotspot, or of its lateral hypothalamic inputs, may produce enhanced “liking” and “wanting”. Colors denote implication in “wanting” (green), fear (red), mixed “wanting” and fear (yellow), suppression of “wanting” or “fear” (gray), “liking” (orange), or suppression of “liking” (blue). All data from sources described in text.

The results of DNQX microinjection studies described earlier indicated that dopamine D1 neurotransmission in NAc was especially crucial for the generation of intense appetitive motivation by AMPA glutamate blockade in medial shell. Selective local D1 blockade prevented the glutamate antagonist microinjection in NAc from producing increases in appetitive eating (e.g., at rostral sites or in a comfortable environment), whereas D2 blockade did not impede such appetitive behavior. By contrast, endogenous D2 stimulation was as necessary as D1 stimulation for the generation of fearful behaviors by the same NAc DNQX microinjections (e.g., at caudal shell sites or in a stressful environment). The unique appetitive contribution of NAc D1-neurons in rats seems consistent with our preliminary optogenetic results in mice, in that both support a special D1 role in appetitive motivation. Selective optogenetic excitation of D1-expressing neurons in NAc generated appetitive motivated behavior in the form of self-stimulation, whereas selective stimulation of D2-expressing neurons did not support self-stimulation. Given that D1-expressing NAc neurons are the only ones belonging to the “direct pathway”, which projects to ventral tegmentum, this pattern again suggests a special D1/direct role in appetitive motivation (see Figure 3). By contrast NAc D2-indirect neurons appear less specifically related to appetitive motivation or self-stimulation reward, and can even be involved in fear. However, we caution that the role of D2 neurons may be complex, as others have reported D2 involvement in appetitive motivation, such as in cocaine self-administration (Bachtell et al., 2005; Loriaux et al., 2011). Also needing further resolution are the relative roles of excitation vs. inhibition of NAc neurons in reward. While results of many studies support the hypothesis that NAc inhibition generally is a chief mechanism of appetitive motivation, it also seems clear that excitation of at least some NAc neurons can similarly support appetitive motivation. Even for D1-direct NAc neurons, DNQX-induced inhibition may generate appetitive eating in rostral NAc shell. Yet, optogenetic-induced excitation appears to generate appetitive self-stimulation throughout the entire NAc shell.

## Conclusion

In summary, hypothalamic-mesocorticolimbic circuitry involving NAc, VP, and LH participates in the control of “wanting” and “liking” for food rewards (Figure 3). Each structure contains functionally distinct subregions, which are revealed by particular manipulations. For example, the NAc shell and VP each contain a smaller hedonic hotspot where opioid and related signals enhance “liking” for food rewards (rostral in NAc; caudal in VP). Each also contains an opioid coldspot that suppresses positive hedonic impact (caudal in NAc; rostral in VP). Likewise, for DNQX/muscimol production of intense motivations in NAc shell, the rostral appetitive zone of the NAc keyboard overlaps with the rostral hedonic hotspot of medial shell. Both of these NAc localizations of function indicate an anatomical bias for appetitive/reward functions within anterior medial shell. Conversely, the caudal NAc zone for DNQX/muscimol production of fearful motivations (and muscimol production of “disgust”) overlaps with the opioid-mediated hedonic coldspot in caudal NAc shell. Both indicate a posterior NAc shell bias for generating negative-valenced motivations and for suppressing reward. Yet, by contrast to these localizations of function to shell subregions, the entire NAc shell (as well as core and regions of neostriatum and amygdala) supports intense appetitive motivation generated by other dopamine, opioid or even optogenetic D1-neuron manipulations. Beyond these specific sites and structures for “liking” and “wanting” functions, it is also recognized that all interact together in larger patterns of mesocorticolimbic circuitry. Considerable insights have emerged from the studies described above, and future research is likely to give further insights into the details of how specific neuronal populations, mechanisms, and hypothalamic-mesocorticolimbic interactions control motivations and the hedonic impact of rewards.

## Conflict of Interest Statement

The authors declare that the research was conducted in the absence of any commercial or financial relationships that could be construed as a potential conflict of interest.
